# Voice acoustics allow classifying autism spectrum disorder with high accuracy

**DOI:** 10.1038/s41398-023-02554-8

**Published:** 2023-07-08

**Authors:** Frédéric Briend, Céline David, Silvia Silleresi, Joëlle Malvy, Sandrine Ferré, Marianne Latinus

**Affiliations:** 1 UMR 1253, iBrain, Université de Tours, INSERM, 37000 Tours, France; 2 University of Milano-Bicocca, Department of Psychology, Milan, Italy; 3 EXAC·T, Centre Universitaire de Pédopsychiatrie, CHRU de Tours, Tours, France; 4 Centro de Estudios en Neurociencia Humana y Neuropsicología. Facultad de Psicología, Universidad Diego Portales, Santiago, Chile

**Keywords:** Diagnostic markers, Human behaviour

## Abstract

Early identification of children on the autism spectrum is crucial for early intervention with long-term positive effects on symptoms and skills. The need for improved objective autism detection tools is emphasized by the poor diagnostic power in current tools. Here, we aim to evaluate the classification performance of acoustic features of the voice in children with autism spectrum disorder (ASD) with respect to a heterogeneous control group (composed of neurotypical children, children with Developmental Language Disorder [DLD] and children with sensorineural hearing loss with Cochlear Implant [CI]). This retrospective diagnostic study was conducted at the Child Psychiatry Unit of Tours University Hospital (France). A total of 108 children, including 38 diagnosed with ASD (8.5 ± 0.25 years), 24 typically developing (TD; 8.2 ± 0.32 years) and 46 children with atypical development (DLD and CI; 7.9 ± 0.36 years) were enrolled in our studies. The acoustic properties of speech samples produced by children in the context of a nonword repetition task were measured. We used a Monte Carlo cross-validation with an ROC (Receiving Operator Characteristic) supervised k-Means clustering algorithm to develop a classification model that can differentially classify a child with an unknown disorder. We showed that voice acoustics classified autism diagnosis with an overall accuracy of 91% [CI95%, 90.40%-91.65%] against TD children, and of 85% [CI95%, 84.5%–86.6%] against an heterogenous group of non-autistic children. Accuracy reported here with multivariate analysis combined with Monte Carlo cross-validation is higher than in previous studies. Our findings demonstrate that easy-to-measure voice acoustic parameters could be used as a diagnostic aid tool, specific to ASD.

## Introduction

Autism spectrum disorder (ASD) is a class of prenatal neurodevelopmental disorders [1] defined by the co-occurrence of two main diagnostic criteria: a socio-emotional impairment and a behavioral deficit manifested by repetitive behaviors and interests [2]. Socio-emotional impairments affect both the production and perception of social signal. To this day, there is no reliable biomarker of ASD, and diagnostic is based on a pluri-disciplinary clinical assessment of the child [3]. Finding a more objective and automated marker of ASD could help in the diagnosis of ASD making it simpler and more reliable [4]. Atypical voice prosody is one of the earliest markers of ASD [5–8], evaluated in diagnostic tools such as ADOS [9]; here, we asked whether easy-to-measure vocal acoustic features could be used as an objective ASD-specific marker to help diagnosis.

The human voice carries a wealth of information regarding a speaker, its physical characteristics, state of mind and health. From birth, the voice is used to signal information on well-being to surrounding adults, and infant cries are part of the preliminary assessment of neonates’ health. Atypical acoustic cry features are associated with central nervous system dysfunction in human neonates [10] and rodent pups [11]. Voice production involves the entire brain and is under the influence of both autonomic and somatic nervous systems [12]. Voice production starts with breathing. The air coming from the lungs is sent towards the larynx, where it induces the vibration of the vocal folds. The vibration of the vocal folds produces a buzzing sound with a particular fundamental frequency, and associated harmonics. This sound is then modulated by its passage through the vocal tract airways. Breathing is normally considered an automatic process, but during speech it can be controlled voluntarily yet unconsciously [12, 13]. Muscles in the larynx are controlled by two different branches of the vagal nerve: the recurrent laryngeal nerve (RLN) and the superior laryngeal nerve (SLN). The RLN controls muscles of the larynx that allow opening, closing, and adjusting the tension of the vocal folds; the SLN allows changing the tension of the vocal folds, therefore increasing fundamental frequency. Articulation of the sound depends on the position of the different elements forming the vocal tract airways and are under voluntary control by the primary motor cortex [e.g., [12]]. Autism spectrum disorder is characterized by impaired functioning of both somatic and autonomic nervous systems, and these impairments have consequences in their vocal production. Consistently, previous studies have reported differences in the acoustic properties of the voice of autistic individuals. Nonetheless results are often contradictory and inconsistent (see [5]). Pitch, measured as the fundamental frequency (f0) of speech sounds, has been reported to be higher in autism [14–18], although many studies do not show this result [19–22]. Jitter, a measure of cycle-to-cycle regularity in f0 frequency, and shimmer, a measure of cycle-to-cycle variation of f0 amplitude, are reported to be smaller in autism with regards to neurotypical individuals [16, 21]; these observations suggest a greater stability of voicing during speech productions in ASD. It should be noted however that other studies report, using different measures, increased pitch variation in autism [14, 15, 22], or lack of differences in pitch variability [23]. Studies of vocal tract features analysis also report discrepant results with higher formant frequencies [18] or a smaller formantic dispersion [16]. These data converge into demonstrating that there is something special in the voice of autistic individuals, that could help in diagnosis. Yet, univariate analysis of specific acoustic measures may not be powerful enough. Here, we describe a multivariate analysis of vocal acoustic parameters combined with machine learning techniques to develop potential tools to aid autism diagnosis.

**Fig. 1:**
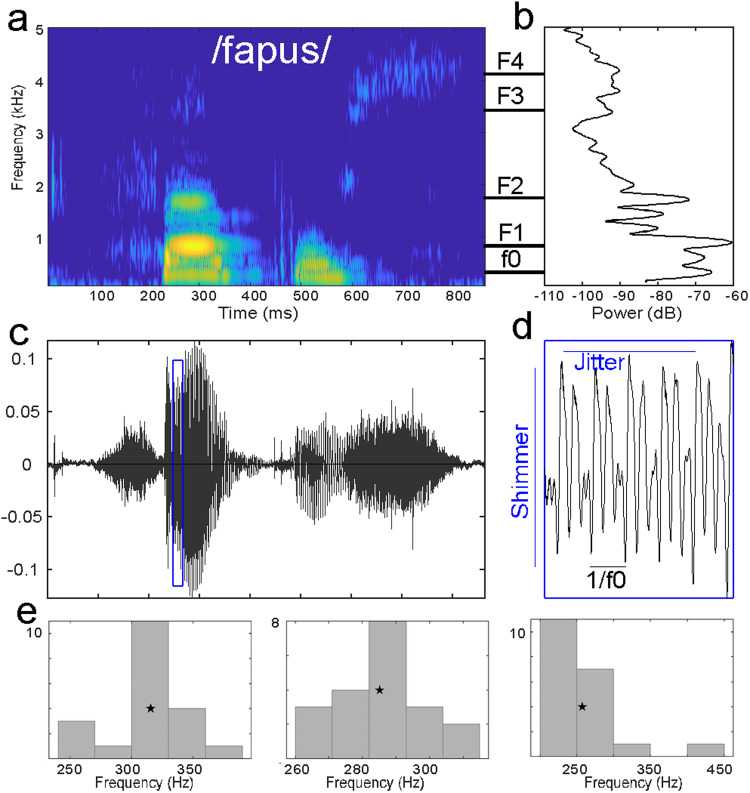
Experimental design. **a** Spectrogram of one of the nonwords produced in the nonword repetition task. **b** Average power spectrum. **c** Amplitude waveform. **d** Zoom on the amplitude waveform to illustrate shimmer and jitter. **e** Distribution of mean f0 measured in the 20 selected nonwords for a skewness (left panel; skewness = 0), and kurtosis (middle panel; kurtosis normalize = 0) corresponding to a normal distribution and for altered (right panel) skewness (3.1) and kurtosis (9.5).

Machine learning techniques are increasingly used for medical diagnosis, especially clustering which is a powerful tool for detecting patterns in datasets. Several studies have used clustering methods in order to develop diagnostic biomarkers of various pathologies in animal models [4, 24, 25] and in human trials [26–28]. A classical, non-supervised, and robust clustering algorithm, the k-Means clustering algorithm [29], also known as nearest centroid classifier when used in machine learning, yields high discriminating power to diagnose a single unknown subject in a given disorder state [25, 27]. Here, we used voice acoustics (Fig. 1) as the selected features included in this common classifier to evaluate their classification performance of ASD relative to typical development and other pathologies. We evaluated the classification performance of voice acoustics in comparison not only to TD children (study 1) but also to children with other disorders sharing common deficits with ASD: sensorineural hearing loss and Developmental Language Disorder (DLD, study 2). These two pathologies were chosen due to observed commonalities in the language domain between children with DLD and autism [30] and between children with sensorineural hearing loss and with cochlear implants (CI) and autism [31]. The acoustic properties of speech samples produced by children in the context of a nonword repetition task (NRT) [32] were examined. Data were analyzed with unsupervised and ROC (Receiving Operator Characteristic) supervised clustering algorithm.

## Methods and Materials

### Participants

**Fig. 2:**
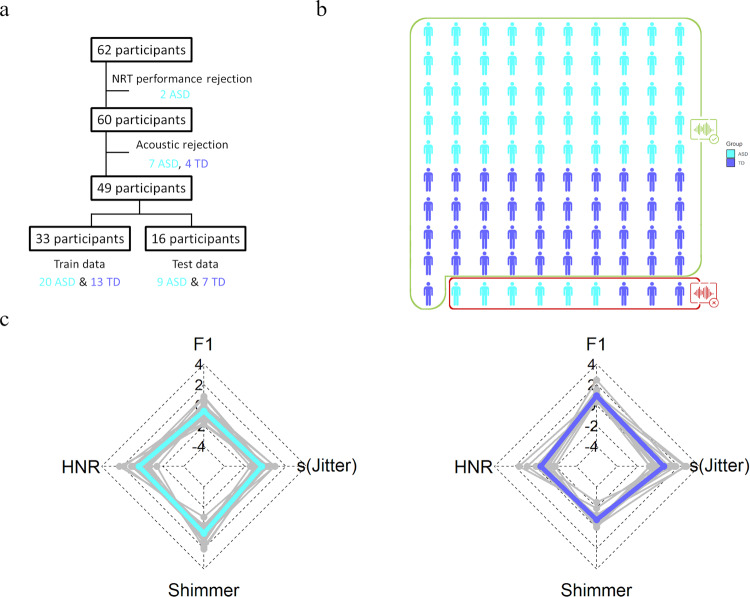
Classification of autistic children with respect to TD children (study 1). **a** Procedure of inclusion of the participants and random dichotomization of the data in diagnostic model group and unknown data group. NRT: Nonword Repetition Task; ASD: Autism Spectrum Disorder/cyan; TD: typically developing children/blue. **b** Extrapolation of the classification on 100 subjects. Participants with good diagnosis are surrounded by a green rectangle (93%), the misclassified by a red one. **c** Illustration of the acoustic profile by radar chart according to the four most significant voice features, namely, harmonic-to-noise ratio (HNR), formant frequencies 1 (F1), skewness of Jitter [s(Jitter)] and Shimmer generated the best ROC-supervised KCA setting; individual data are displayed in gray.

**Fig. 3:**
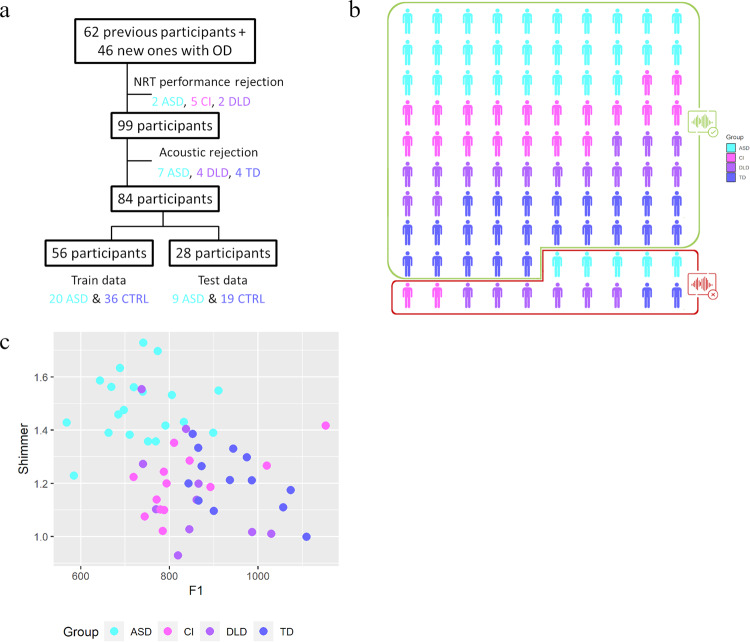
Classification of autistic children with respect to a diverse control population (study 2). **a** Procedure of inclusion of the participants and random dichotomization of the data in diagnostic model group and unknown data group. OD: Other Disorders; NRT: Nonword Repetition Task; ASD: Autism Spectrum Disorder/cyan; CTRL: heterogeneous control group (composed of children with developmental language disorder [DLD], violet, and cochlear implant, pink [CI]). **b** Extrapolation of the classification on 100 subjects. Participants with good diagnosis are surrounded by a green rectangle (84%), the misclassified by a red one. **c** The top two most significant voice features, namely, formant frequencies 1 (F1) and Shimmer generated the best ROC-supervised KCA setting are plotted against each other.

One hundred and eight children were enrolled in our retrospective studies. Study 1 (Fig. 2a) is composed of 38 children on the autism spectrum (1 girl; 8.5 ± 0.25 years) and 24 TD children (12 girls; 8.2 ± 0.32 years), and Study 2 (Fig. 3a) additionally includes 21 children with DLD (9 girls; 7.9 ± 0.51 years) and 25 children displaying severe-to-profound sensorineural hearing loss fitted with CIs (8 girls; 8 ± 0.22 years). Data of 24 children were excluded from the analysis (see Experimental protocol and data acquisition). Therefore, the final sample comprised 84 children distributed as follows: 29 ASD (0 girl; 8.4 ± 0.29 years; age range [6.3 12]; ADOS severity score: 6.19 ± 0.45; CARS: 27.7 ± 0.7), 20 TD (10 girls; 7.99 ± 0.33 years; age range [6 10.5]), 20 CI (6 girls; 8.2 ± 0.19 years; age range [6.5 9.9]; 12 with bilateral CI; 6 with right CI; 2 with left CI; age at first implantation 1.86 ± 0.15) and 15 DLD (7 girls; 8.2 ± 0.37 years; age range [6.5 10.8]). Demographic and clinical information regarding the final samples are presented in Table 1. Youth with ASD received an expert clinical diagnosis based on Diagnostic and Statistical Manual of Mental Disorders – fifth Edition – (DSM-V) [33]; the Autism Diagnostic Interview-Revised [34], and/or the Autism Diagnostic Observation Schedule [35] were used by experienced clinicians of the Excellence Center of Autism (Exac·t), Tours, France to inform diagnostic decisions. Children with DLD also received an expert clinical diagnosis based on the DSM-V [33] Nonverbal cognitive abilities were assessed either by Raven Progressive Matrices or Block Design and Matrix Reasoning of the WISC-IV (data of 5 TD children are missing). Only children with a minimum Mean Length of Utterances of 2.5 were included in the study [36] to ensure that language tests could be administered.

**Table 1:** Demographic and clinical information of the sample.

	N	Male/Female	Age (years)	FRI ± sem *(Percentile)*	RPM ± sem *(Percentile)*	NRT Score ± sem *(%)*
*ASD*	29	29 / 0	8.42 ± 0.29	88.6 ± 5.56 (22)	50.7 ± 13.8 (7)	80 ± 3.1
*TD*	20	10 / 10	7.99 ± 0.33	121.7 ± 4.43 (15)		96 ± 0.92
*DLD*	15	8 / 7	8.15 ± 0.27	93 ± 3.14 (11)	36.9 ± 10.7 (4)	49 ± 6.3
*CI*	20	14 / 6	8.16 ± 0.16	96.6 ± 4.26 (20)		42 ± 4.6

Numbers in brackets represent the number of participants contributing data to the measure. *FRI* Fluid Reasoning Index calculated with the prorate sum of Block Design and Matrix Reasoning scores in WISC-IV, *RPM* Raven’s Progressive Matrices, *NRT* Nonword Repetition Task, *ASD* Autism Spectrum Disorder, *TD* Typically developing children, *DLD* children with Developmental language disorder and *CI* children with Cochlear Implant.

This study was carried out in accordance with the recommendations of the local ethics committee (Comité de Protection des Personnes [CPP] Tours Ouest 1, n°2006-RS), with written informed consent from all parents of the children and assent from the children, in accordance with the Declaration of Helsinki.

### Experimental protocol

Acoustic data were extracted from 20 speech samples recorded in the context of a nonword repetition task [32], therefore reducing the influence of social interaction in voice production. The task focuses on complex phonological structures that have been identified as the source of errors in children with impaired phonology. Briefly, children had to repeat 50 or 70 nonwords of varying phonological complexity, presented with a computer either with only auditory or with both audio and visual information. Nonwords were created using 1, 2 or 3 of the three most common vowels among the languages of the world, namely [a], [i], [u], and from a concise list of consonants which included two stops ([k], [p]), two fricatives ([f], [s]), one liquid [l]. Nonwords had a maximum of 3 syllables to limit the influence of working memory on the repetition task. Nonwords had different syllable complexity: either a simple consonant vowel syllables, syllables with a final consonant or syllables with initial and median consonant clusters [32]. Phonological analysis of the data presented in the current manuscript are published elsewhere [37, 38]. Among the 50 or 70 nonwords, the 20 ones with less phonological errors were chosen for acoustical analysis (see the audio material of non-words retained in the study on the OSF platform). The NRT took place in a quiet room and audio were digitally recorded using Zoom H4 microphones put on a table in front of the child. Overall performance in the NRT task is presented in the results section and was analyzed with a 1 factor Welch ANOVA.

### Acoustic measurements

Acoustic parameters were analyzed using the open-source software Praat [39]. For each nonword, we extracted 9 acoustics parameters (Fig. 1): mean fundamental frequency (f0), mean formant frequencies (F1 to F4), mean formant dispersion (FD), mean harmonic-to-noise ratio (HNR), mean jitter (cycle-to-cycle variation in frequency of f0) and mean shimmer (cycle-to-cycle variation in intensity of f0).

Start and end of the non-word were identified visually, and average values of the frequency parameters (f0, and formant values) were measured on the total length of the non-word. To measure f0, a Pitch object was created with the following parameters: time step = 0.01 s, pitch floor = 90 Hz; pitch ceiling = 600 Hz. To measure formant frequencies, a Formant object using the Burg method was created with the following parameters: time step = 25% of window length, maximum number of formants = 5, maximum formant = 6500 Hz; window length = 0.025, pre-emphasis from 50 Hz. Formant dispersion was calculated as the average difference between formants.

Evaluation of periodicity-related parameters were performed on the 50% central part of the non-word, that is on an interval staring at the start point plus 25% of non-word duration and ending at the end point minus 25% of non-word duration. Mean HNR was measured on the Harmonicity object based on a forward cross-correlation analysis; the Harmonicity object was created with the default parameter values except for minimum pitch which was set to 90 Hz. Jitter and shimmer were measured on the Point process (periodic, cross-correlation) object with 90 Hz and 600 Hz as minimum and maximum pitch, respectively. Jitter is measured as the relative average perturbation using default parameter values (e.g., shortest period = 0.0001, longest period = 0.02, maximum period factor = 1.3). Shimmer (local, dB) was measured as the average absolute base-10 logarithm difference between the amplitudes of consecutive periods, multiplied by 20 with the same parameters than jitter and 1.6 as the maximum amplitude factor.

Parameters were then averaged across the 20 nonwords. In addition, because ASD is characterized with increased intra-individual variability [22, 40, 41], shape parameters (e.g., skewness and kurtosis) of f0, FD, HNR, jitter and shimmer were computed using Matlab2018b functions, leading to 19 variables (Fig. 1). Note that in the Matlab kurtosis function, the normal distribution has a kurtosis value of 3 (Fig. 1e).

Acoustic data are excluded according to two categories of rejection criteria: the nonword repetition task performance and acoustic rejection. For the first criteria, children whose performance in the repetition of vowels was considered outlier ([Q1-1.5xIQR] with Q1: lower quartile and IQR: interquartile range) were removed from the analysis (*N* = 9: 2 ASD, 2 DLD, 5 CI), to avoid bias due to the mispronunciation of certain vowels which can influence acoustics. For the second criteria, based on acoustical analysis (recording quality or outlier value of acoustic parameters with respect to the population), another 15 children (7 ASD; 4 DLD; 4 TD) were excluded from the analysis (ROC-supervised k-Means classification results including all participants but those with poor recordings quality are shown in Supplementary table 2).

### Development of clustering diagnostic model

Code used in this manuscript is available on osf (https://osf.io/veqpz/). Our goal was to determine if acoustic features of the voice could be used as a feature classification specific for autism by k-Means classifying ASD against typical and other atypical development, thus we randomly dichotomized data in a diagnostic model group (train set) and an unknown data group (test set). To validate our model performance, we used a Monte Carlo cross-validation algorithm. Note that this method is robust to imbalance gender across groups; indeed, with a clustering approach, if gender were an important factor the two identified clusters would reflect gender separation rather than diagnostic group.

To develop the diagnostic model, within the Monte Carlo cross-validation, we randomly selected n ASD and n control as train data (70% of data) to which we applied a k-Means clustering algorithm (50 iterations, Hartigan & Wong algorithm); this was repeated 500 times with random subsampling of the data from the entire population, e.g., Monte Carlo cross-validation. Because there is no general rule regarding the number of repetitions to use, we choose the value at which our main criteria (selectivity and sensitivity) appear stable beyond reasonable doubt, through multiple testing with different numbers of resampling (Supplementary Fig. 1). The number of clusters was set to two, since we aimed to determine ASD diagnostic against a control population (TD children only, or control children). We performed k-Means clustering analysis (KCA) in an unsupervised way with the nine acoustic and derived acoustic variables (*N* = 19) and assessed its performance. Then, in order to enhance our KCA, we performed ROC as proposed by Nikas and colleagues ^25^and used an AUC (Area Under the Curve) ROC curve, as measure of separability to evaluate the most discriminative acoustic parameters. This latter probability is an assessment of the discriminative power of a given variable with respect to two measures, here the two groups involved. For example, with a given variable, an AUC of 1 is synonym of a separation between groups with 100% accuracy, and the given variable is considered as a perfect classifier. On the contrary, the worst discrimination between the two groups has an AUC = 0.50 (i.e., no discrimination capacity). In this way, the ROC curve allows us to optimize our KCA by supervising it using acoustic variables with the best discriminative performance. We used a threshold of AUC > 0.80 (80%) corresponding to a good discrimination [42].

This model was then tested to identify the diagnostic group of the test data (30% of the entire data, corresponding to the data not used in model building) according to their KCA classification, for each of the 500 bootstrap replications. To realize this, test data were added one-by-one for each participant and classified by supervised and unsupervised KCA. Hence, diagnostic of the participant was classified based on its data; accuracy was measured as the total number of correct classifications over the total number of classifications.

To assess the performance of our KCA, we measured selectivity, sensitivity and the classification performance of our model. Moreover, goodness of fit, an evaluation of clustering efficiency and of KCA quality, was assessed using the percent of variation (PV), a measure of corresponding to the total within-cluster sum of squares by the total of within and between-cluster sum of squares. Mean values derived from the 500 repetitions linked to the Monte Carlo cross-validation and their associated standard errors are reported for percent variation, selectivity, and sensitivity. Mean values and confidence intervals (95%CI) of classification accuracy was derived from the Monte Carlos cross-validation algorithm.

## Results

### Classification of autistic children with respect to TD children

Overall performance in the NRT task, measured on the entire set of items, differed between TD children (95.6%; performance range [84 100]) and children with ASD (80.4%; performance range [28 100]; Welch ANOVA: F(1, 31.7) = 22.3; *p* < 0.001).

To investigate the discriminative power of voice acoustics between autistic children and TD children, we performed K-Means clustering analysis (KCA). Data were randomly split into a train group (*N* = 33; 20 ASD, 13 TD) and a test group (unknown data; *N* = 16; 9 ASD; 7 TD); this was done 500 times. Unsupervised KCA with the 19 acoustics variables was conducted on the train group, and the model was cross validated using repeated random sub-sampling using unknown data from the test group. We observed a percent of variation (PV, the dispersion between the two clusters; see Methods) of 83.20% ± 1.33, a sensitivity of 0.74 ± 0.11 and a specificity of 0.92 ± 0.12 in the training group. The unsupervised KCA correctly classified 73.1% [71.8% 74.4%] of ASD and 92.3% [91.4% 93.1%] of TD children (Fig. 2b).

Our goal was to find optimum KCA settings, which best separate the ASD and the TD group to develop classification or diagnostic model. Therefore, we performed ROC curve analysis, to conduct ROC-supervised KCA [25, 43] on training and test data with 500 bootstrap replications. The four most discriminant (Area Under the Curve [AUC] > 80%) acoustics parameters according to ROC analysis on the training group were mean F1, mean HNR, mean shimmer and jitter skewness. The ROC-supervised KCA setting yielded a considerable improvement over unsupervised KCA: as shown in Fig. 2, the ROC-supervised KCA had a PV of 63.85% ± 3.00, a sensitivity of 0.89 ± 0.06 and a specificity of 0.94 ± 0.10; it classified correctly 89% [88.1% 90.0%] of ASD and 93% [92.7% 94.3%] of the TD group. ROC supervision resulted in a decrease of false negatives.

### Classification of autistic children with respect to a control population

Overall performance in the NRT task, measured on the entire set of items, was affected by diagnostic group (Welch ANOVA: F(3, 33.3) = 62.1; *p* < 0.001). It was higher for TD children than all other groups (all pairwise comparisons *p* < 0.001). Autistic children performed better than SLI (49.3% [6 76]) and IC children (42.3% [10 80]; all pairwise comparisons *p* < 0.001), who did not differ.

Next, we evaluated the classification performance of voice acoustics in comparison not only to TD children but also to children with other disorders sharing common deficits with ASD: sensorineural hearing loss and Developmental Language Disorder. As previously described, we conducted a ROC-supervised KCA on the data of all participants, considering children with DLD, children with CI and TD children in the same group of heterogeneous control group (CTRL). See Supplementary table 1 for details about the unsupervised KCA.

Two acoustic parameters discriminated ASD from CTRL children according to ROC analysis: mean shimmer and F1, with an AUC, respectively of 85.23% and 82.36% (the separation is depicted in the Fig. 3c). The ROC-supervised KCA had a PV of 57.42% ± 3.04, a sensitivity of 0.86 ± 0.05 and a specificity of 0.84 ± 0.08; it classified correctly 85.56% [84.5%-86.6%] of ASD and 84.2% [83.5%-85.0%] of the CTRL group. More specifically, in this latter heterogeneous population, 68.3% [66.8%-70.5%] of DLD, 85.6% [84.4%-86.8%] of CI and 93.9% [93.1%-94.7%] of TD were correctly classified (Fig. 3b).

## Discussion

### Voice as clustering diagnostic approach?

As classifier, the ROC-supervised KCA analysis, with classification performance around 90%, had an extremely high classification performance when separating ASD from TD children above previously reported classification value (between 80 and 89% [4, 6, 22]). Moreover, our method proves robust and reliable in discriminating autistic children from children without ASD, including other disorders (84%).

Importantly, acoustic factors predictive of autism diagnosis are mainly ones related to control of the vocal folds’ vibrations (e.g., jitter, shimmer) rather than the *f0* per se [5], consistent with clinical description of a peculiar voice quality in autism and previous observations [23]. The pattern characteristics of autism, with respect to TD children, was lower average F1, higher HNR, higher shimmer and lower jitter skewness. A lower jitter skewness reflected a more normal distribution of jitter across nonwords, consistent with the observation of a greater stability in voice production [16]; TD children presented positively skewed and less tailed distribution, highlighting that most vocal sounds had similar shimmer and jitter. Note that we found that children on the autism spectrum have a higher shimmer than NT children, contrarily to what was reported in adults [16], highlighting differences in the maturation of the vocal apparatus. A higher HNR suggested that vocal sounds of children on the autism spectrum are overall less noisy than those produced by TD children. The lower F1 in the ASD group did not reflect gender balance differences across groups as in the TD group male and female children had similar F1 values (938 Hz and 937 Hz). Note that when including the other pathologies, only mean F1 and mean shimmer remained classification features. Average F1 values were at the minimum 100 Hz lower in autistic children than in the other children; this is unlikely explained by gender imbalance as the second lowest F1 was observed for female (815 Hz) of the CI group, and F1 was the highest in male of the same group. F1 frequency is related to the length of the vocal tract [44] and tend to decrease with age; a lower F1 could reflect either an accelerated maturation of the vocal tract or differences in cranio-facial anatomy [45] and the presence of increased minor physical anomalies in autistic children [46]. Shimmer, which is a measure of cycle-to-cycle variation in amplitude of the *f0*, presented an increased value of almost 16% in autism; although discriminant as in Guo et al., [6], the opposite result was found in children speaking mandarin. Shimmer differences could reflect morphological differences or differences in control of the vocal cords of autistic children and other children [47]. Therefore, these voice features could be an external marker of atypical neurodevelopment occurring before birth [1]. Note that in the current study we aimed to test whether voice could be used as a classification tool, and therefore we tested older children with stable diagnostic. Future studies should aim at studying the classification performance of infant’s cries at birth or within the first year of life to test the validity of voice acoustics as a true biomarker of autism.

Previous studies that compared ASD to other populations are sparse and rely on different grouping strategies: Oller et al. [48] reported 62% accuracy in the classification of children with DLD in a non-TD group, while Bone et al. [49] reported a 78% of correct classification between ASD and DLD. Here, classifying children with DLD in the typically developing group, we obtained high classification rates of ASD not only with respect to TD, but also to other pathologies. The approach developed here combining feature selection, through ROC-supervision, a clustering analysis and Monte Carlo cross-validation demonstrates that voice features have a strong, specific, diagnosis power for ASD: accuracy was well-above chance for children with DLD and children with CI. This provides new information on the classifying power of voice features in ASD, in relation to other neurodevelopmental disorders in particular (e.g., DLD).

Central nervous system dysfunction affects vocal folds and by domino individual’s voice. This is why automated voice analysis using recordings of patient speech is increasingly being used in psychiatry [50] and neurology as digital biomarkers of disease (i.e., in Major depressive disorder [51], schizophrenia [52], Parkinson’s disease [53], Alzheimer’s Disease [54], …). However, this computational method should not be delegated solely to machines [55], even if it is based on formal reasoning, this method should be used in complementarity to clinical diagnosis of experts.

The current pilot study is a proof-of-concept towards the development of an early diagnostic biomarker specific to ASD. Yet, the sample size of the group used are very small and need to be much larger to define an established clinical biomarker. Futures studies should aim at replicating this result with considerably larger sample sizes. Moreover, in order to develop a sensitive diagnosis test, future works should include typical cases met in clinical practice, with disorders more often seen as comorbidities of ASD such as attention deficit hyperactivity disorder (ADHD), motor problems without social impairment, severe anxiety, and other behavior disorders [56]. In this study, all children had at some minimum, strong verbal capabilities and data were selected to have the most optimum dataset; future studies should assess the classification performance of vocal acoustic based on non-linguistic vocal samples acquired in less controlled environment. In addition, data presented here comes from children between 6 and 12 while, in high-income countries the average age of ASD diagnosis is around age 4 [56], and around 5 worldwide [57]. To be truly a biomarker of autism and understand its potential diagnostic value, these results should be replicated in younger children and possibly using cry features of babies.

## Conclusion

Overall, our work suggests that easy-to-measure voice features, potentially linked to abnormal early neurodevelopment, can help in the diagnosis of autism spectrum disorder. Voice features in supervised clustering methods can be used as a potential feature classification for autism and paves the way to a new objective tool to aid clinical and differential diagnosis of ASD. The method developed here is in part automated, and in the future, a hand-in tool should be developed to automatically output diagnostic information. Early detection of ASD is crucial because it is likely to lead to an improved outcome. Thus, based on our simple clustering algorithm method, future work should investigate the acoustic cry features of baby as a potential biomarker for autism.

## Supplementary information

Supplementary Figures and Table

## Data Availability

All data generated and/or analyzed during this study are available from the corresponding author (M.L.) on reasonable request. For all clustering runs, we used R (http://cran.r-project.org).
