# Supplementary material for: Voice acoustics allow classifying autism spectrum disorder with high accuracy
Source: Transl Psychiatry. 2023 Jul 8;13:250. doi: 10.1038/s41398-023-02554-8 (PMC10329669; doi:10.1038/s41398-023-02554-8)
Supplement: Supplementary file 1 — Supplementary Figures and Table [file 41398_2023_2554_MOESM1_ESM.docx]

# Supplemental information

|  | KCA | | |
| --- | --- | --- | --- |
|  | Unsupervised (19 variables) |  | ROC-supervised (2 variables) |
| *Diagnostic model group (20 ASD vs 36 CTRL)* | |  |  |
| Sensitivity (mean ± sd) | 0.78 (0.08) |  | 0.86 (0.05) |
| Specificity (mean ± sd) | 0.72 (0.11) |  | 0.84 (0.08) |
| (+)Likelihood Ratio (mean ± sd) | 0.63 (0.10) |  | 0.77 (0.09) |
| (-)Likelihood Ratio (mean ± sd) | 0.86 (0.05) |  | 0.91 (0.02) |
| Percent of variation (mean ± sd) | 86.79 (0.81) |  | 57.42 (3.04) |
|  |  |  |  |
| *Unknown data group (9 ASD vs 19 CTRL)* |  |  |  |
| Sensitivity (mean ± sd) | 0.78 (0.07) |  | 0.86 (0.04) |
| Specificity (mean ± sd) | 0.72 (0.09) |  | 0.84 (0.08) |
| (+)Likelihood Ratio (mean ± sd) | 0.62 (0.09) |  | 0.76 (0.09) |
| (-)Likelihood Ratio (mean ± sd) | 0.86 (0.04) |  | 0.92 (0.02) |
| Percent of variation | 86.84 (0.77) |  | 57.48 (2.88) |
| Classification accuracy % Correct [CI95%] |  |  |  |
| Total | 73.89 [73.15-74.61] |  | 84.66 [84.05-85.26] |
| 9 ASD | 77.82 [76.57-79.02] |  | 85.56 [84.49-86.57] |
| 19 CTRL | 72.03 [71.11-72.93] |  | 84.24 [83.49-84.96] |
|  |  |  |  |

**Table S1: Summary of the results of N = 500 Monte-Carlo cross validation KCA after rejecting participants who were outliers in either nonword repetition task performance, or acoustic measurements and those who had poor quality recordings.** Each clustering method was tested both in an unsupervised and ROC-supervised way. The percent of variation of a clustering method assesses the clustering ﬁtting of that method in a given setting. ASD: Autism Spectrum Disorder participants; CTRL: heterogeneous control group (composed of children with developmental language disorder [DLD] and cochlear implant [CI]); CI95%: Confidence interval of 95%. Data are presented as mean (Standard Deviation).

|  | KCA | | |
| --- | --- | --- | --- |
|  | Unsupervised (19 variables) |  | ROC-supervised (2 variables) |
| *Diagnostic model group (22 ASD vs 44 CTRL)* | |  |  |
| Sensitivity (mean ± sd) | 0.81 (0.07) |  | 0.83 (0.05) |
| Specificity (mean ± sd) | 0.57 (0.10) |  | 0.79 (0.10) |
| (+)Likelihood Ratio (mean ± sd) | 0.49 (0.07) |  | 0.69 (0.11) |
| (-)Likelihood Ratio (mean ± sd) | 0.86 (0.05) |  | 0.91 (0.03) |
| Percent of variation (mean ± sd) | 86.51 (0.77) |  | 60.42 (2.51) |
|  |  |  |  |
| *Unknown data group (11 ASD vs 22 CTRL)* |  |  |  |
| Sensitivity (mean ± sd) | 0.81 (0.06) |  | 0.83 (0.05) |
| Specificity (mean ± sd) | 0.57 (0.09) |  | 0.79 (0.09) |
| (+)Likelihood Ratio (mean ± sd) | 0.49 (0.06) |  | 0.69 (0.10) |
| (-)Likelihood Ratio (mean ± sd) | 0.86 (0.04) |  | 0.90 (0.03) |
| Percent of variation | 86.51 (0.73) |  | 60.45 (2.39) |
| Classification accuracy % Correct [CI95%] |  |  |  |
| Total | 65.83 [65.10-66.65] |  | 79.92 [79.29-80.52] |
| 11 ASD | 81.53 [80.47-82.54] |  | 83.36 [82.35-84.34] |
| 22 CTRL | 57.98 [57.05-58.90] |  | 78.19 [77.40-78.95] |
|  |  |  |  |

**Table S2: Summary of the results of N = 500 Monte-Carlo cross validation KCA with all data except those with poor recording quality.** Each clustering method was tested both in an unsupervised and ROC-supervised way. The percent of variation of a clustering method assesses the clustering ﬁtting of that method in a given setting. ASD: Autism Spectrum Disorder participants; CTRL: heterogeneous control group (composed of children with developmental language disorder [DLD] and cochlear implant [CI]); CI95%: Confidence interval of 95%. Data are presented as mean (Standard Deviation).


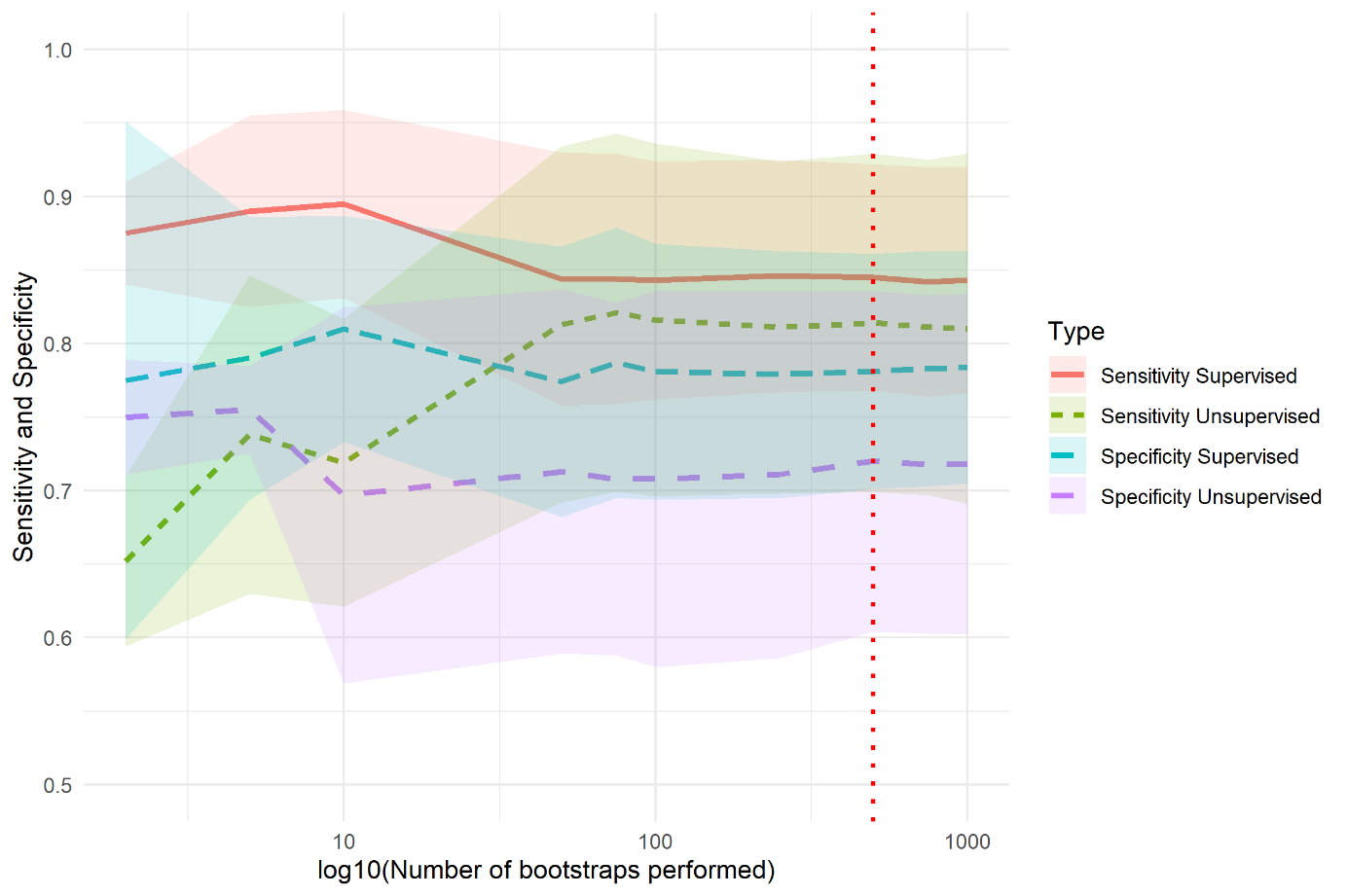


**Supplementary Fig. 1. Number of bootstraps for K-means clustering Analysis decided by convergence criteria.** The number of bootstrap samples that it takes to get a stable histogram is the optimal, increasing bootstraps will not change the sensitivity and specificity. Here for the study 2, 500 bootstrap iterations were used and materialized by red vertical dotted line.
